# Cooperative Control for A Hybrid Rehabilitation System Combining Functional Electrical Stimulation and Robotic Exoskeleton

**DOI:** 10.3389/fnins.2017.00725

**Published:** 2017-12-21

**Authors:** Dingguo Zhang, Yong Ren, Kai Gui, Jie Jia, Wendong Xu

**Affiliations:** ^1^State Key Laboratory of Mechanical Systems and Vibrations, Robotics Institute, Shanghai Jiao Tong University, Shanghai, China; ^2^Huashan Hospital, School of Medicine, Fudan University, Shanghai, China

**Keywords:** knee exoskeleton, functional electrical stimulation, hybrid rehabilitation, cooperative control, central pattern generator

## Abstract

Functional electrical stimulation (FES) and robotic exoskeletons are two important technologies widely used for physical rehabilitation of paraplegic patients. We developed a hybrid rehabilitation system (FEXO Knee) that combined FES and an exoskeleton for swinging movement control of human knee joints. This study proposed a novel cooperative control strategy, which could realize arbitrary distribution of torque generated by FES and exoskeleton, and guarantee harmonic movements. The cooperative control adopted feedfoward control for FES and feedback control for exoskeleton. A parameter regulator was designed to update key parameters in real time to coordinate FES controller and exoskeleton controller. Two muscle groups (quadriceps and hamstrings) were stimulated to generate active torque for knee joint in synchronization with torque compensation from exoskeleton. The knee joint angle and the interactive torque between exoskeleton and shank were used as feedback signals for the control system. Central pattern generator (CPG) was adopted that acted as a phase predictor to deal with phase confliction of motor patterns, and realized synchronization between the two different bodies (shank and exoskeleton). Experimental evaluation of the hybrid FES-exoskeleton system was conducted on five healthy subjects and four paraplegic patients. Experimental results and statistical analysis showed good control performance of the cooperative control on torque distribution, trajectory tracking, and phase synchronization.

## 1. Introduction

Neurologic injuries such as stroke and spinal cord injury may cause paresis in patients and give rise to movement disability. Physical rehabilitation is highly necessary for paralyzed individuals to restore mobility of extremities. Functional electrical stimulation (FES) and robotic exoskeletons are two important technologies used widely in extremity rehabilitation.

Many FES systems have been developed by using either surface or implanted electrodes in the past decades (Popovic et al., 2001). As a neuro-rehabilitation approach that excites and activates muscles directly, FES can provide not only functional training but also therapeutic benefits to paralyzed patients. Although some advances in closed-loop control and multichannel selection of muscles have achieved complex stimulation, it is still a complicated and tough problem of controlling FES to assist paralyzed individuals to move in a natural manner, mainly due to the nonlinearity and time variability of human musculoskeletal system (Zhang et al., 2007; Lynch and Popovic, 2008). The pathological muscle conditions and the poor controllability of FES result in insufficient joint torque to provide limbs movement and body support for patients (del Ama et al., 2012; Ha et al., 2012; Quintero et al., 2012). In addition, muscle fatigue is often induced under continuous electrical stimulation. In a word, these problems mentioned severely hinder the widespread usage of FES from becoming a popular treatment option.

Robotic exoskeleton is an alternative technology of extremity rehabilitation for paraplegic patients, and lower limb exoskeletons are designed to accomplish neuro-rehabilitation and replace the physical gait training effort of therapists (Dollar and Herr, 2008). The well-known representatives in the application of motor rehabilitation for lower limbs are Lokomat (Hocoma, Switzerland) (Colombo et al., 2000), LOPES (Veneman et al., 2007), POGO and PAM (Reinkensmeyer et al., 2006), ALEX (Banala et al., 2009), etc. The popular exoskeletons usually use electric actuators, hydraulic actuators, or pneumatic actuators (Fan and Yin, 2013; Vitiello et al., 2013). In comparison with FES, the therapeutic effect of robotic rehabilitation is limited, because it can merely provide assistive torque to limbs, the muscles are not stimulated actively, which are passively contracted or stretched. Therefore, it is an urgent demand to combine FES with exoskeletons, merging as hybrid rehabilitation systems that bring about not only functional but also physiological benefits to patients.

There is an increasing interest in developing hybrid rehabilitation systems, taking the advantages of FES and exoskeleton, and overcoming the limitations in separate application (To et al., 2008; del Ama et al., 2012). In general, there are two kinds of such hybrid rehabilitation systems, i.e., combination of FES and powerless (passive) orthoses, or combination of FES and powered (active) exoskeletons. The controlled-brake orthosis (CBO) developed by Goldfarb and Durfee (1996) used joint brakes to control the body movement generated by FES. An obvious deficiency of orthoses is the inability to generate active torque for joints. Compared with orthoses, powered exoskeletons using mechanical actuators can compensate insufficient torque generated by FES. Recently, some achievements in hybrid FES-exoskeleton systems have been made, such as WalkTrainer (Stauffer et al., 2009), Vanderbilt Exoskeleton (Ha et al., 2012), Kinesis (del Ama et al., 2014), iLeg (Chen et al., 2014) and so on. In WalkTrainer system, Stauffer et al. (2009) developed closed-loop control of FES that modulated muscle stimulation to minimize the interaction force between the wearer and the exoskeleton, or modulated the desired torques as a function of the gait cycle. That system did not take account for muscle fatigue compensation as the exoskeleton was not actively involved. In order to accomplish cooperative control of FES with the Vanderbilt Exoskeleton during walking, Ha et al. (2016) proposed a two-loop controller, where motor control loop and muscle control loop co-existed. In that manner, the motor control loop used joint angle feedback to control the output of the joint motor to track the desired joint trajectories, while the muscle control loop utilized joint torque profiles from previous steps to regulate the muscle stimulation for the subsequent step to minimize the motor torque contribution required for joint angle trajectory tracking. del Ama et al. (2014) proposed cooperative control to balance the effort between muscle stimulation and exoskeleton in hybrid system (Kinesis), which sought to minimize the interaction torque and realized hybrid ambulatory gait rehabilitation. The torque-time integral generated by FES was measured to estimate muscle fatigue and a learning method was used to modulate the stimulation strength so as to compensate the torque loss. Alibeji N. A. et al. (2015) and Alibeji et al. (2017) developed an adaptive control method inspired by muscle synergy to compensate for actuator redundancy and FES-induced muscle fatigue in a hybrid FES-exoskeleton system, which showed ability to coordinate FES of quadriceps and hamstrings muscles and electric motors at the hip joint and knee joint of the exoskeleton. Chen et al. (2014) designed an FES-assisted control strategy for a hybrid lower-limb rehabilitation system (iLeg), where active FES control was achieved via a combination of neural network based feedforward control and PD feedback control to realize torque control, and meanwhile impedance control was adopted for exoskeleton control. Tu et al. (2017) combined FES with exoskeleton to accomplish gait rehabilitation in a different way, where FES and exoskeleton made effect on different joints separately, i.e., exoskeleton was applied on hip and knee joints, and FES was applied on ankle joint. A sliding control algorithm called chattering mitigation robust variable control (CRVC) was used for cooperative control in that hybrid system.

This study aims to accomplish harmonic and elegant control between FES and exoskeleton and explore their combined function on single-joint movement. Different from previous works, the active roles of FES and exoskeleton can be set freely here, i.e., the contribution of FES and exoskeleton can be distributed arbitrarily under different circumstances with specified requirements. Meanwhile, the synchronization problem of different drivers (motor vs. muscle) is well solved. It is well known knee joints play very important roles in lower limb locomotion, and knee joint control is a benchmark in previous literature (Chang et al., 1997; Ferrarin et al., 2001; Hunt et al., 2004; Sharma et al., 2009; Alibeji N. et al., 2015). Therefore, a hybrid rehabilitation system called FEXO Knee is developed in this work, which combines FES with a knee exoskeleton. A novelty of the system is the interactive force can be measured, which can help realize the better cooperative control. Moreover, it is very interesting and challenging to synchronize the human leg (driven by biological muscles) and exoskeleton (driven by artificial motor) to accomplish one task together, which is particularly solved in this work. A new cooperative control scheme is proposed, which can achieve shank swing motion under the harmonized and synchronized action of FES and exoskeleton, and realize different contribution of FES and exoskeleton. In such a scheme, a biologically-inspired control method, central pattern generator (CPG), is adopted because CPG has some favorable properties in synchronization, entrainment, and robustness against disturbance in general (Ijspeert, 2008). A combination of feedforward control and feedback control is used for FES and exoskeleton. A parameter regulator based on policy gradient method is designed to coordinate FES controller and exoskeleton controller adaptively. Five healthy subjects and four hemiplegic patients have participated in a series of experiments to test the cooperative control performance of FEXO Knee.

## 2. Method

### 2.1. FEXO knee

**Figure 1:**
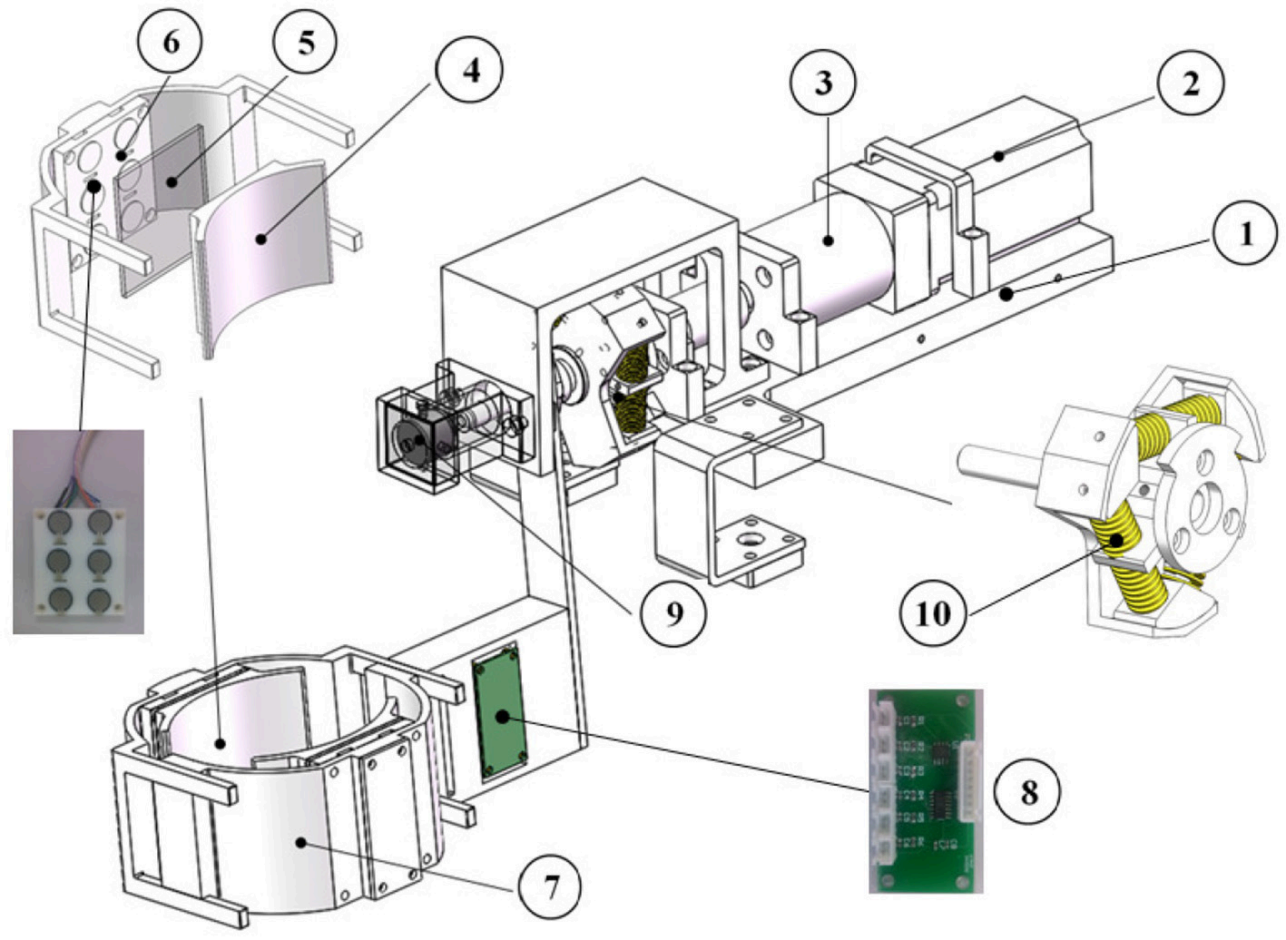
Structure of exoskeleton in FEXO Knee: (1) base bench, (2) electric motor (AC servo motor), (3) reducer, (4) shank wrap, (5) silicone board, (6) interactive force sensors, (7) outer shell, (8) signal amplification circuit, (9) encoder, (10) linear springs.

The cooperative control of FES and exoskeleton is accomplished on our available prototype, FEXO Knee, which has two parts: a self-designed knee exoskeleton and a commercial FES device (RehaStim 2, Hasomed, Germany). The exoskeleton is composed of mechanical parts, electric motor, elastic actuator, sensors, and accessories. The function of exoskeleton is to generate assistive torque for rhythmic swing of human shank. It is designed for subjects with sitting posture, so it has a base bench that may be fixed on a table to hold the whole structure. The preliminary version (FEXO Knee I) has been reported in Ren and Zhang (2014). The new version (FEXO Knee II) is shown in Figure 1.

#### 2.1.1. Mechanical design and actuation

The main mechanical frame of the knee exoskeleton is made of aluminum. The key part is the electric motor (i.e., an AC servo motor of Panasonic Corp., Japan), which has a maximum angular velocity of 5,000 rpm, 400 W rated power, and a nominal torque of 1.3 Nm. The planetary reducer combined with the motor has speed radio of 15:1, thus the output end of the reducer can generate a nominal torque of 19.5 Nm.

The output shaft of the reducer connects to an elastic component via a rigid coupler. The elastic component consists of six linear springs with a stiffness of 16.5 N/mm. The six springs with pre-contraction are placed between a three-spoke element and an output fixture (see Figure 1). The torque generated by the servo motor can be transmitted to the output fixture through a rotatory elastic module, which turns into a series elastic actuator (SEA) (Pratt and Williamson, 1995; Tsagarakis et al., 2009). For the whole elastic component, the stiffness is a variable and can be given by:

(1) $$\begin{array}{r} K_{SEA}=6\cdot K_{A}\cdot \left(R^{2}+\frac{r_{s}^{2}}{3}\right)\cdot \left(2\cos\vartheta_{s}^{2}-1\right) \end{array}$$

where *K*_*SEA*_ denotes the stiffness of the elastic component; *K*_*A*_ denotes the stiffness of a single linear spring; *R* denotes the spoke radius; *r*_*s*_ denotes the external radius of a single linear spring; ϑ_*s*_ is the net rotatory angle, which is the difference between motor output angle and actual exoskeleton rotation angle ($\vartheta_{s}=\vartheta_{m}-\vartheta_{e}^{a}$). According to the design size of the knee exoskeleton, we know *R* = 0.027 m, *r*_*s*_ = 0.008 m.

The mechanism that holds the human shank is fastened to the output fixture of the elastic component, and contains two adjustable shells. Two interactive force sensors are placed between the outer shell and the shank wrap. For safety purpose, the range of motion (ROM) of the joint is limited to ±90° for knee extension and flexion. The naturally drooping state of human shank is defined as the zero position.

#### 2.1.2. Sensors

Two types of sensors are installed in the knee exoskeleton: an absolute encoder for measuring the joint angular position, and two interactive force sensors for measuring the mutual force between exoskeleton and shank. The encoder is fastened coaxially with the joint with resolution of 0.09°. The two interactive force sensors are respectively attached to the front and the rear of the shell, and please refer to component (6) in Figure 1. Each contains six distributed force sensing resistors (FSR 402, Interlink Electronics, USA) covered with a silicone board. The total interactive force is the summation of measured data from six calibrated FSR elements. The mutual torque (τ_*mut*_) between exoskeleton and shank can be obtained through multiplying the interactive force by the force arm. According to the mechanical structure, the average force arm is 0.16 m for the knee exoskeleton. The mutual torque is defined as positive if it is acted on shank in extension direction and negative in flexion direction. For real-time control, FEXO Knee uses a data acquisition card (USB-6343, National Instrument, USA) to receive signals from these sensors, and the sampling frequency is 1 Hz. In fact, the interactive force sensors should be a highlight of this system, which can measure force variation generated by muscles (e.g., force decline due to muscle fatigue), and provide important information for cooperative control.

### 2.2. Control scheme

**Figure 2:**
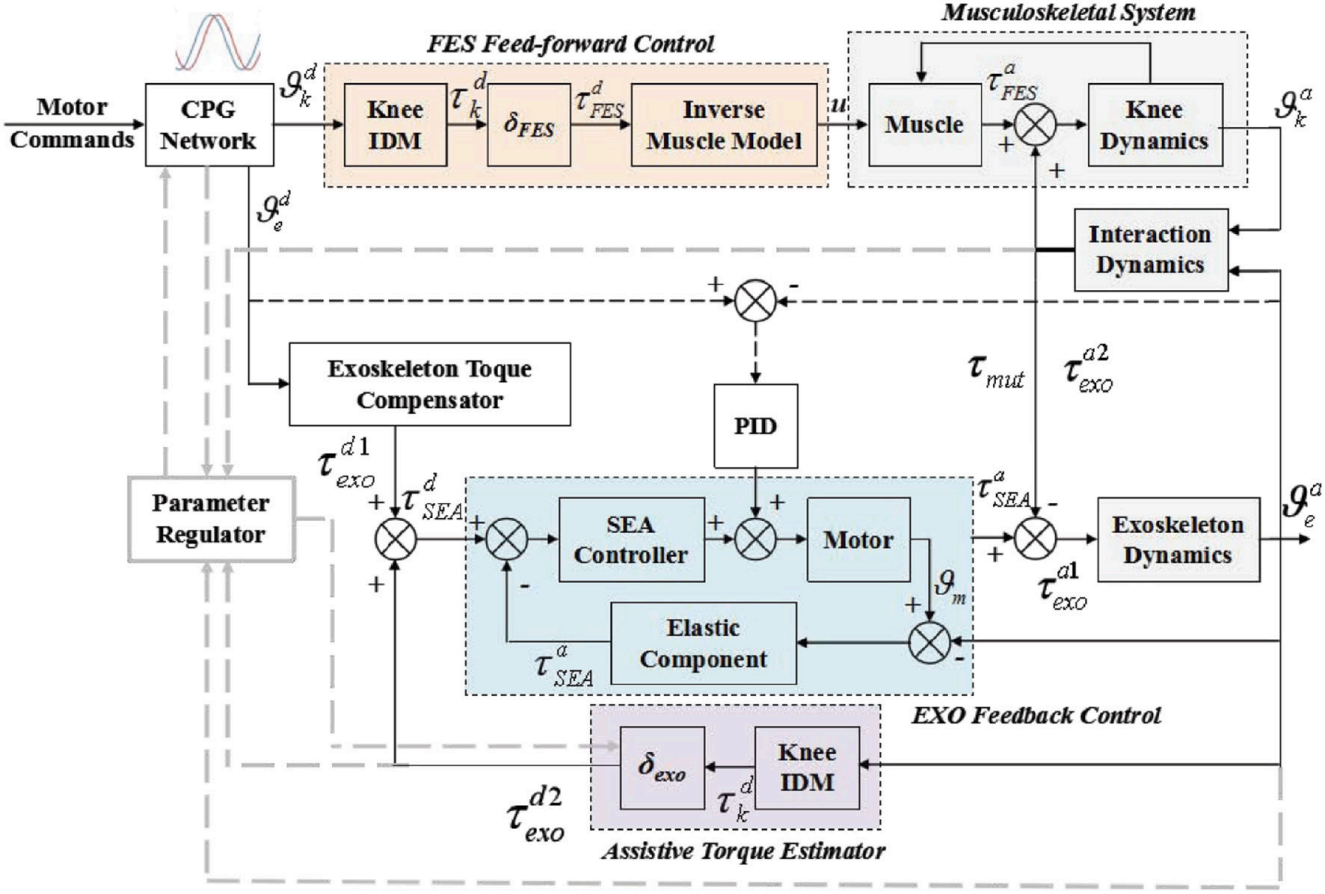
Block diagram of cooperative control scheme for FES and exoskeleton in a hybrid rehabilitation system (FEXO Knee). The controlled plant includes knee musculoskeletal system and exoskeleton. The feedfoward control strategy is used for FES, and the feedback control strategy is for exoskeleton. CPG network and parameter regulator are in charge of synchronization of FES and exoskeleton.

In the FEXO Knee system, two different kinds of actuators (skeletal muscles and electric motor) should work together. The cooperative control is the kernel, which aims to achieve suitable synchronization and compliant interaction between the knee exoskeleton driven by electric motor and the human shank activated by FES. The control scheme of FEXO Knee is shown in Figure 2. The desired total torque ($\tau_{k}^{d}$) for knee joint movement is supplied by summation of desired FES torque ($\tau_{FES}^{d}$) and desired assistive torque from exoskeleton ($\tau_{exo}^{d2}$). The torque distribution between them is regulated by two tunable gains (δ_*exo*_ and δ_*FES*_). In fact, the exoskeleton should generate two parts of desired torque, compensating its own dynamics ($\tau_{exo}^{d1}$), and contributing to knee joint movement ($\tau_{exo}^{d2}$). In our system, the total torque output of exoskeleton is realized via SEA ($\tau_{SEA}^{d}$), which is the summation of $\tau_{exo}^{d1}$ and $\tau_{exo}^{d2}$. In practice, the actual output of SEA minus the actual torque for exoskeleton dynamics ($\tau_{exo}^{a1}$) is the actual assistive torque for knee joint from exoskeleton ($\tau_{exo}^{a2}$), which is measured by the interactive force sensors and indicated by τ_*mut*_. Therefore, τ_*mut*_ is the same as $\tau_{exo}^{a2}$.

The overall control architecture is mainly composed of four parts: (1) the reference trajectory generator based on the CPG model, (2) the feedfoward controller for modulating pulse width of FES, (3) the feedback controller of the knee exoskeleton used to compensate the insufficient part of torque generated by FES, and (4) the parameter regulator with online adaptive updating rules for key parameters.

#### 2.2.1. CPG network

According to previous research in neurophysiology, CPGs have been demonstrated to be a kind of neural control mechanism in central nervous system of animals, which can generate rhythmic locomotion independently. In addition, CPGs have some inherent advantages of entrainment, synchronization, robustness, which are desired features in the cooperative control of rhythmic movements. A variety of mathematical models have been developed to simulate the CPG function in a simplified form, which are widely used in robotic control, e.g., Matsuoka oscillators (Zhang and Hashimoto, 2012), Hopf oscillators (Righetti et al., 2006), and phase oscillators (Farzaneh and Akbarzadeh, 2012), etc. In this work, we use a phase oscillator, which was firstly adopted by Ijspeert et al. to control a salamander-like robot (Ijspeert et al., 2007). To make the model match our requirement, the original form of the phase oscillator is modified. Two mutually coupled nonlinear oscillators form the CPG network. The modified oscillator model is given by the following equations:

(2) $$\begin{array}{r} \dot{\phi_{i}}\left(t\right)=\omega_{i}\left(t\right)+\overset{2}{\underset{j}{\sum }}\nu_{ij}\sin\left(\phi_{j}\left(t\right)-\frac{\omega_{j}}{\omega_{i}}\phi_{i}\left(t\right)-\Phi \right) \end{array}$$

(3) $$\begin{array}{r} \ddot{\alpha_{i}}\left(t\right)=\mu_{\alpha i}\left[\frac{\mu_{\alpha i}}{4}\left(A_{i}-\alpha_{i}\left(t\right)\right)-\dot{\alpha_{i}}\left(t\right)\right] \end{array}$$

(4) $$\begin{array}{r} \ddot{\omega_{i}}\left(t\right)=\mu_{\omega i}\left[\frac{\mu_{\omega i}}{4}\left(\Omega_{i}-\omega_{i}\left(t\right)\right)-\dot{\omega_{i}}\left(t\right)\right] \end{array}$$

where ϕ_*i*_, α_*i*_, and ω_*i*_ are state variables, and denote the phase, amplitude and frequency of a nonlinear oscillator respectively; Φ is a variable that denotes the desired phase difference between two oscillators; *A*_*i*_ and Ω_*i*_ denote the desired amplitude and frequency of a oscillator respectively; μ_α*i*_ and μ_ω*i*_ are some positive parameters that represent the velocity of a oscillator transformed into a new locomotion state; ν_*ij*_ is a positive parameter that denotes the coupling weight between two oscillators. For all these parameters, the subscribe *i* = 1, 2.

In general, CPG has three basic output features (phase, amplitude, and frequency), which can be freely set based on requirement. This model uses nonlinear differential equations to realize CPG, which can generate smooth and stable trajectories even during transitional periods between different patterns. The output of a single nonlinear oscillator in CPG can be represented by:

(5) $$\begin{array}{r} \vartheta_{i}\left(t\right)=\alpha_{i}\left(t\right)\sin\left(\phi_{i}\left(t\right)\right) \end{array}$$

where ϕ_*i*_ is a state variable and denotes the phase of the *i*th oscillator, ϑ_*i*_ denotes the output trajectory of the *i*th oscillator. Here, the two outputs ϑ_1_ and ϑ_2_ serve as the desired trajectories for knee joint ($\vartheta_{k}^{d}$) and exoskeleton ($\vartheta_{e}^{d}$).

Besides, the velocity and acceleration of the output trajectory can be conveniently obtained as follows:

(6) $$\begin{array}{r} \frac{d^{k}\vartheta_{i}}{dt^{k}}\left(t\right)=\alpha_{i}\omega_{i}^{k}\sin\left(\phi_{i}+\frac{\pi }{2}k\right). \end{array}$$

Therefore, the desired angle, angular velocity and acceleration for knee joint ($\vartheta_{k}^{d},{\dot{\vartheta }}_{k}^{d},{\ddot{\vartheta }}_{k}^{d}$) and the exoskeleton ($\vartheta_{e}^{d},{\dot{\vartheta }}_{e}^{d},{\ddot{\vartheta }}_{e}^{d}$) can be generated by the two coupled oscillators.

The reference joint angle, angular velocity and acceleration generated by CPG can be used to estimate the desired torque. Besides, the phase difference between reference trajectories of FES and the exoskeleton (Φ) is one of the most important parameters in the control scheme, which can be online regulated to avoid possible confliction between human shank and knee exoskeleton. The desired motion commands (*A*_*i*_, Ω_*i*_, etc.) for different motor patterns are adjusted manually based on experimental protocol.

#### 2.2.2. Feedfoward control for FES

The feedfoward controller for FES contains three parts: (1) an inverse dynamics module, in which the inputs are the joint angle, angular velocity and acceleration provided by the CPG network, and the output is desired actuation torque of human knee joint; (2) a torque distribution gain δ_*FES*_, which represents the percentage of torque that FES should provide; (3) an inverse muscle model, which is used to obtain the modulated stimulation pulse width as the output of FES.

An inverse dynamics model (IDM) of the knee joint movement (shank swing) is developed, which is used to calculate the desired torque in the feedfoward controller:

(7) $$\begin{array}{r} \tau_{k}^{d}\left(t\right)=I_{s}\ddot{\vartheta }\left(t\right)+B_{s}\dot{\vartheta }\left(t\right)+K_{s}\vartheta \left(t\right)+mgl_{s}\sin\vartheta \left(t\right) \end{array}$$

where *I*_*s*_[Nms^2^/rad], *m*[kg] and *l*_*s*_[m] are the segment (shank and foot) inertia, mass and equivalent length, respectively; *B*_*s*_[Nms/rad] and *K*_*s*_[Nm/rad] are the knee viscous damping and stiffness coefficients; ϑ(*t*)[rad], $\dot{\vartheta }\left(t\right)$[rad/s] and $\ddot{\vartheta }\left(t\right)$[rad/s^2^] denote the knee angle, angular velocity and acceleration, using the desired trajectory and its first and second derivatives (i.e., $\vartheta_{k}^{d},{\dot{\vartheta }}_{k}^{d},{\ddot{\vartheta }}_{k}^{d}$); *g* = 9.81 m/s^2^ is the gravity constant; $\tau_{k}^{d}\left(t\right)$[Nm] denotes the total desired torque needed for knee joint.

The desired torque that should be generated by human muscles under electrical stimulation is a fraction of the estimated total torque obtained by IDM, i.e., $\tau_{FES}^{d}=\delta_{FES}\cdot \tau_{k}^{d}$, and the fraction is determined by the FES distribution gain δ_*FES*_. An inverse muscle model based on Hill-type musculotendon actuator is used to acquire the pulse width of FES. The Hill-type model illustrates the activation and contraction dynamics of human muscles. A model-based control method is adopted, which used a piecewise linear recruitment function to describe the muscular activation dynamics, a Gaussian function to describe the torque-angle relation, and a linear function to approximate the torque-angular velocity relation (Ferrarin et al., 2001). The inverse muscle model can be given by:

(8) $$\begin{array}{rcl} a\left(t\right) & = & \tau_{FES}^{d}\left(t\right)\cdot \exp\left\{{\left(\frac{\vartheta \left(t\right)+\pi /2-\lambda_{1}}{\lambda_{2}}\right)}^{2}\right\}\cdot {\left(1-\lambda_{3}\dot{\vartheta }\left(t\right)\right)}^{-1} \end{array}$$

(9) $$\begin{array}{rcl} u\left(t\right) & = & \frac{a\left(t\right)\left(u_{sat}-u_{thres}\right)}{u_{sf}}+u_{thres} \end{array}$$

where *a*(*t*)[Nm] denotes the muscle activation; λ_1_[rad], λ_2_[rad] and λ_3_[rad^−1^s] are muscle and joint specific parameters used in the contraction dynamics; *u*(*t*)[μs] is the stimulation pulse width; *u*_*sat*_[μs], *u*_*thres*_[μ] and *u*_*sf*_[μ] denote, respectively, the threshold, the saturation, and the scaling factor. These parameters are individual variables, which can be acquired through experimental indentification.

#### 2.2.3. Feedback control for exoskeleton

The exoskeleton should provide two parts of torque: one is to support its own motion, and the other is for human knee joint motion. The exoskeleton torque compensator is designed firstly, in which the knee exoskeleton is considered independently without regard to the interaction with human leg. The desired driven torque ($\tau_{exo}^{d1}$) for the exoskeleton dynamics itself is estimated through an impedance model given by:

(10) $$\begin{array}{r} \tau_{exo}^{d1}=I_{e}{\ddot{\vartheta }}_{e}^{d}+B_{e}{\dot{\vartheta }}_{e}^{d}+K_{e}\vartheta_{e}^{d} \end{array}$$

where *I*_*e*_, *B*_*e*_, and *K*_*e*_ denote the inertia, viscous damping and stiffness of the exoskeleton, which are obtained by system identification in experiment. $\vartheta_{e}^{d}$ is the desired trajectories of exoskeleton provided by CPG. The human leg would not bear any burden from the exoskeleton if $\tau_{exo}^{d1}$ is completely generated by electric motor in this case.

The desired assistive torque ($\tau_{exo}^{d2}$) that should be provided by the exoskeleton depends on a distribution gain δ_*exo*_, which is a fraction of total torque of knee joint, i.e., $\tau_{exo}^{d2}=\delta_{exo}\cdot \tau_{k}^{d}$. We design δ_*exo*_ and δ_*FES*_ as a pair of distribution gains. In theory, δ_*exo*_ + δ_*FES*_ = 1. However, it has some difference in practice, because δ_*FES*_ is a fixed parameter, while δ_*exo*_ is a flexible parameter that is updated online by parameter regulator. The total torque of knee joint is also calculated according to the knee IDM Equation (7), but it should be acquired in a real-time approximation for feedback control, which needs the information of actual angular position, velocity and acceleration. In fact, the raw angle data measured by an encoder always contain noise signals, which would make the estimation very rough if we directly use the derivatives of the joint angle. In our control system, a state estimation method based on adaptive phase oscillators proposed by Ronsse et al. is used to acquire the angular position, velocity and acceleration (Ronsse et al., 2011, 2013). Thus, a relatively smooth estimation of total torque can be obtained. The product of the estimated total torque and the gain δ_*exo*_ is the desired assistive torque $\tau_{exo}^{d2}$ that the exoskeleton should provide. In ideal condition, $\tau_{exo}^{d2}$ should be equal to mutual torque τ_*mut*_. In sum, the desired torque of SEA ($\tau_{SEA}^{d}$) contains two parts, one is for masking the dynamics of the exoskeleton and the other is for providing necessary assistive torque.

The SEA controller realizes the torque control by a proportional-derivative (PD) method, and the control parameters $k_{SEA}^{p}$ and $k_{SEA}^{d}$ are tuned at 0.1 and 0.001 by trial and error. The mutual torque between human leg and the exoskeleton (τ_*mut*_) is measured in real time, which represents the actual assistive torque for human leg or the resistant torque for the exoskeleton. Therefore, the actual output torque of SEA ($\tau_{SEA}^{a}$) minus the mutual torque (τ_*mut*_) is the actual exoskeleton torque ($\tau_{exo}^{a1}$), which drives the exoskeleton itself.

Besides, a classical proportional-integral-derivative (PID) controller is implemented as a position controller to reduce the trajectory tracking error, and make the motion of exoskeleton smooth and accurate. The error signal is the difference between the reference trajectory and the actual angle of exoskeleton. The control parameters *k*_*p*_, *k*_*i*_, and *k*_*d*_ are tuned at 6.0, 0.12, and 0.01 by trial and error in the experiment. Even though the closed-loop PID is for angular position control, the absolute accuracy of trajectory tracking is not the most important issue in the control paradigm. Actually, the combination of SEA torque control and PID position control allows compliant interaction between the knee exoskeleton and the human leg, as well as appropriate trajectory tracking of knee joint.

#### 2.2.4. Parameter regulator

Parameter regulator is the key part in the cooperative control scheme, which aims to update two key parameters, Φ (cf. Equation 2) and δ_*exo*_. Parameter regulator has three pairs of inputs (desired and actual angle, desired and actual angular velocity, desired and actual mutual torque), and two outputs (Φ and δ_*exo*_). The two outputs are the online adaptive parameters. Please note angular velocity is not measured directly from sensors, and it is achieved by derivative operation of angle. Therefore, only two pairs of inputs are shown in the parameter regulator in Figure 2. Φ is for CPG, which can provide synchronization for FES and exoskeleton. δ_*exo*_ is for exoskeleton controller, which can make exoskeleton adaptively compensate the inadequate torque from FES (e.g., muscle fatigue).

Due to variations of different individuals in different situations, an online regulating strategy based on policy gradient methods is used to adjust these parameters (Kaelbling et al., 1996; Peters and Schaal, 2006). FEXO Knee mainly focuses on rhythmic locomotion, thus every period can be considered as a task trial. A fitness function is introduced to assess the motion performance:

(11) $$\begin{array}{r} J=\int_{t_{0}}^{t_{f}}\left(0.125e_{\tau }^{2}+1.2e_{p}^{2}+0.556e_{v}^{2}\right)dt \end{array}$$

where *t*_0_ and *t*_*f*_ represent the beginning and end of a trial; *e*_*p*_, *e*_*v*_ and *e*_τ_ denote the position error of knee joint ($\vartheta_{k}^{d}-\vartheta_{k}^{a}$), the angular velocity error (${\dot{\vartheta }}_{k}^{d}-{\dot{\vartheta }}_{k}^{a}$), and the assistive torque error ($\tau_{exo}^{d2}-\tau_{exo}^{a2}$), respectively. Actually, the mutual toque (τ_*mut*_) is equal to $\tau_{exo}^{a2}$. The updating rule of relevant parameters can be given by:

(12) $$\begin{array}{rcl} \delta_{exo,h+1} & = & \delta_{exo,h}-\gamma_{\delta }\nabla_{\delta_{exo}}J{|}_{\delta_{exo}=\delta_{exo,h}} \end{array}$$

(13) $$\begin{array}{rcl} \Phi_{h+1} & = & \Phi_{h}-\gamma_{\Phi }\nabla_{\Phi }J{|}_{\Phi =\Phi_{h}} \end{array}$$

where γ_δ_ and γ_Φ_ denote learning rates and *h* ∈ {0, 1, 2, …} is the updating number. The updating time window is three cycle periods (trial durations) for the two parameters, Φ and δ_*exo*_, because of the partial derivatives in the discrete gradient descent method.

## 3. Experiments and results

Experiments were conducted on nine subjects for evaluating the performance of FEXO Knee with the cooperative control method proposed. The experiments were approved by the Ethics Committee of Shanghai Jiao Tong University, China. All subjects were volunteers and signed the informed consent before the experiments.

### 3.1. Subjects

Five healthy subjects (H1~H5) and four patients (P1~P4) participated in the experiments. The basic information of the nine subjects is shown in Table 1. All the five healthy subjects had no history of neurological or muscular disease. All the four patients were hemiplegic. P1~P3 had paralysis on right side, and P4 had paralysis on left side. The disease time of the four patients was all less than 10 months. The experiments on the hemiplegic patients were conducted in Shanghai Huashan People's Hospital.

**Table 1:** Information of subjects.

**Subj**.	**Gender**	**Age**	**Height [cm]**	**Mass [kg]**	**Physical condition**
H1	Male	24	168	61	Healthy
H2	Male	25	165	55	Healthy
H3	Male	24	170	65	Healthy
H4	Male	25	168	59	Healthy
H5	Male	25	170	73	Healthy
P1	Female	35	160	63	Brain Injury
P2	Male	61	160	70	Stroke
P3	Male	66	170	75	Stroke
P4	Male	64	156	58	Stroke

Some parameters in IDM for each subject were estimated according to the anthropometric calculation method in Winter (2009). The mass of the segment (shank and foot) was estimated as 6.1% of the total body weight, the length as 28.5% of the total body height, the center of mass as 60.6% of the segment length, and the radius of gyration as 73.5% of the segment length. The inertia is calculated as the product between the segment mass and the square of the segment radius of gyration, i.e., *I* = *m*(0.735*l*)^2^[Nms^2^/rad]. Other parameters in IDM were estimated according to the empirical method in Ferrarin et al. (2001). This is a rough model of the actual knee dynamics, so the variations in stiffness *K* and the damping coefficient *B* during knee flexion and extension movement were not considered. The movement range of knee joint angle is −35° ~ +35°. The stiffness and damping coefficients were calculated by the equations: *K* = ω^2^*I*−*mgl*/2[Nm/rad], *B* = 2ηω*I*[Nms/rad] (Lin and Rymer, 1991). In experiments, η and ω of IDM were respectively tuned at 0.5 and 6 Hz for all subjects for simplicity, resulting an under-damped knee motion.

### 3.2. Experimental setup and protocol

Before the evaluation experiments, some parameters about the control system of FEXO Knee should be preset, which were grouped for CPG network, FES feedfoward controller, exoskeleton feedback controller, and parameter regulator.

The parameters of CPG network were determined by user requirement and literature (Ijspeert et al., 2007). The parameters for exoskeleton feedback controller were determined by simple system identification in experiments. The parameters for FES feedfoward controller were determined by experimental methods in the reference (Ferrarin et al., 2001), and some pilot tests on subjects. The parameters of regulator were determined by trial-and-error test and literature (Peters and Schaal, 2006). The general parameters for all the subjects are shown in Table 2. The subject-dependent parameters are shown in Table 3. Regarding parameter setting of FES, the pulse frequency was set at 50 Hz for all the subjects, the pulse amplitude *I*_*a*_ [mA] was a subject-dependent parameter, and the pulse width was the only controlled variable.

**Table 2:** Common control parameters.

**Parameter**	**Value**	**Parameter**	**Value**	**Parameter**	**Value**
ν_12_	2.0	μ_α1, 2_	5.0	μ_ω1, 2_	5.0
γ_δ_	10^−5^	γ_Φ_	10^−5^	μ_*sf*_ [μs]	15
λ_1_[rad]	0.87	λ_2_[rad]	1.13	λ_3_[rad^−1^s]	0.04
$I_{e}\left[kgm^{2}\right]$	0.04	*B*_*e*_[Nms/rad]	2.0	*K*_*e*_[Nm/rad]	3.50

**Table 3:** Subject-specific stimulation parameters.

**Subj**.	**$\mu_{sat}^{HAM}$ [μs]**	**$\mu_{sat}^{QUA}$ [μs]**	**$\mu_{thres}^{HAM}$ [μs]**	**$\mu_{thres}^{QUA}$ [μs]**	***I*_*a*_ [mA]**
H1	400	350	50	50	25
H2	400	370	50	50	30
H3	450	380	50	50	30
H4	380	370	50	50	25
H5	400	400	50	50	25
P1	260	260	100	100	30
P2	400	400	100	100	35
P3	420	420	100	100	40
P4	420	420	100	100	40

*HAM, hamstrings; QUA, quadriceps*.

**Figure 3:**
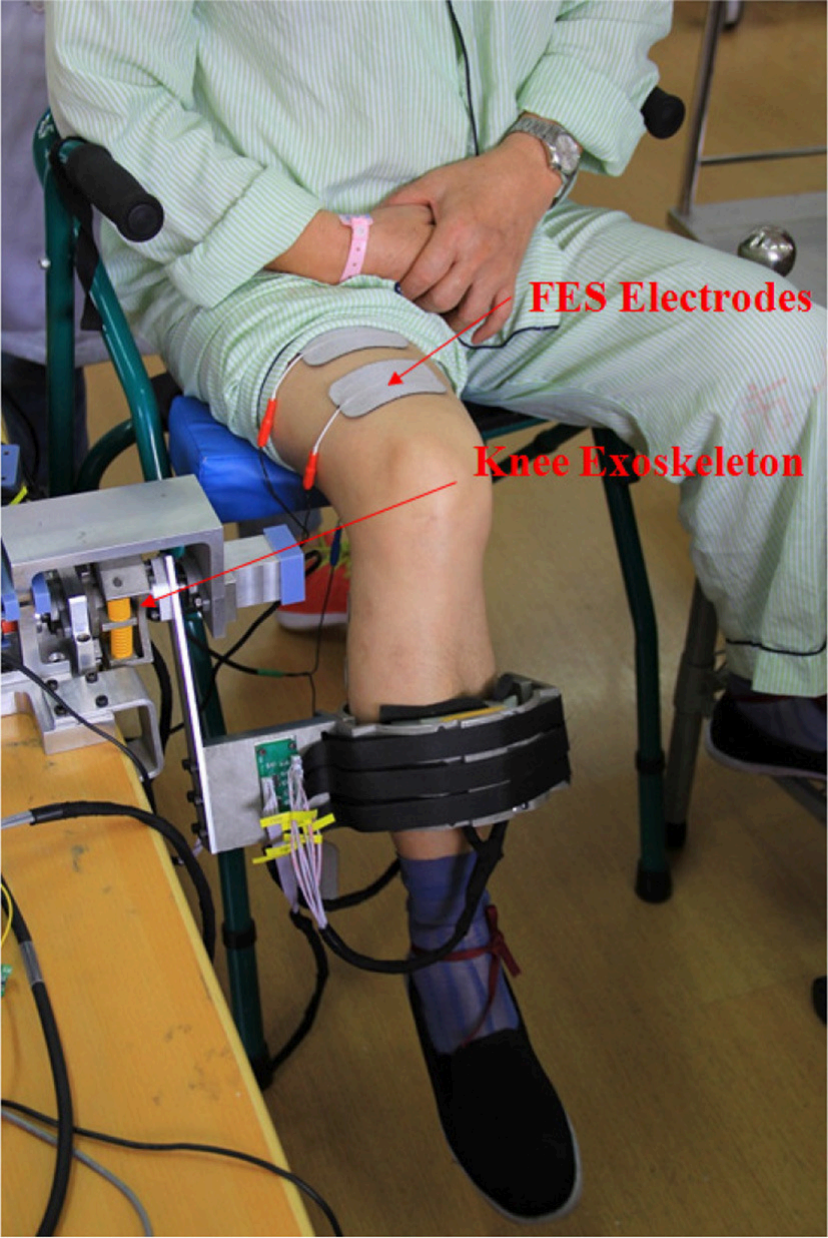
Experimental setup of hybrid FES-exoskeleton rehabilitation system (FEXO Knee). A paralyzed patient (P3) wearing FEXO Knee was taking experiment, where outer shell of exoskeleton held the shank, and FES electrodes were attached to skin over targeted muscles (quadriceps and hamstrings).

During experiment, the subject sat on a chair and wore FEXO Knee on the leg, two-channel FES surface electrodes were placed over the anterior and posterior thigh, targeting two muscle groups (quadriceps and hamstrings), as shown in Figure 3. The subject was told not to perform any voluntary movements during the experimental procedure.

**Figure 4:**
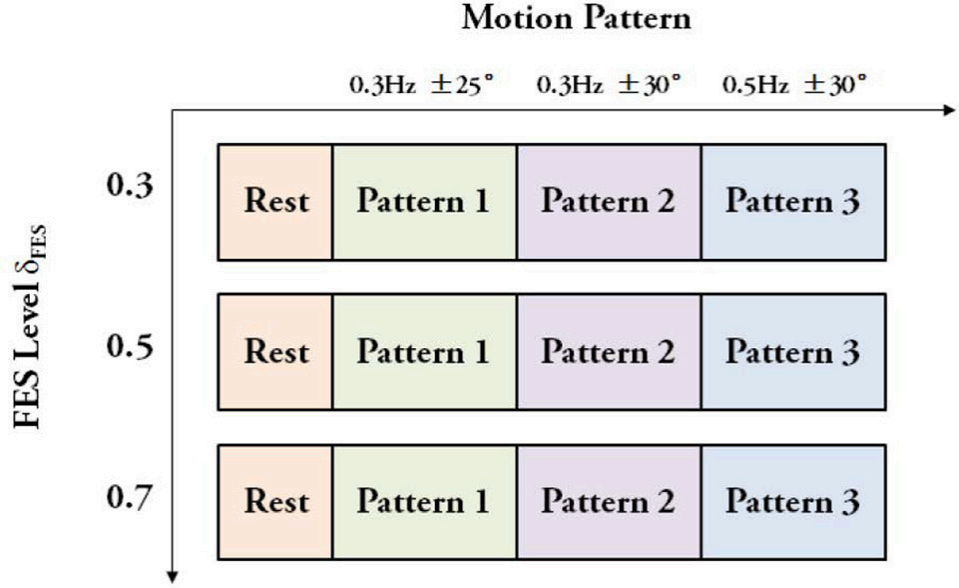
Experimental protocol. Three sessions with different FES levels were conducted, and each session had three motion patterns.

The evaluation experiments were designed to assess the cooperative control performance of FEXO Knee under different FES levels. The purpose is to check if exoskeleton can provide the proper assistive torque for knee joint if FES makes different contribution. The experimental protocol is shown in Figure 4. In the experiments, each subject accomplished three sessions according to the FES level based on distribution gain (δ_*FES*_). The distribution gain was arbitrarily chosen as δ_*FES*_ = 0.3, 0.5, 0.7, meaning that the torque provided by FES accounted for 30, 50, and 70% of the total joint torque. In each session, the reference trajectories generated by the CPG module provided three kinds of motion patterns: Pattern 1–movement frequency 0.3 Hz and maximum angular amplitude ±25°; Pattern 2–movement frequency 0.3 Hz and maximum angular amplitude ±30°; Pattern 3–movement frequency 0.5 Hz and maximum angular amplitude ±30°. There were breaks between the sessions for the subjects to rest. For healthy subjects, each motion pattern continued for 120 s. Considering the lower endurance of paralyzed patients, their experimental duration was a little shorter. The whole procedure of the experiments was carried out by the control software of FEXO Knee. During the steady state of evaluation experiments, no subjects reported confliction or disturbance between leg and exoskeleton using the FEXO Knee system.

### 3.3. Data processing and results

**Figure 5:**
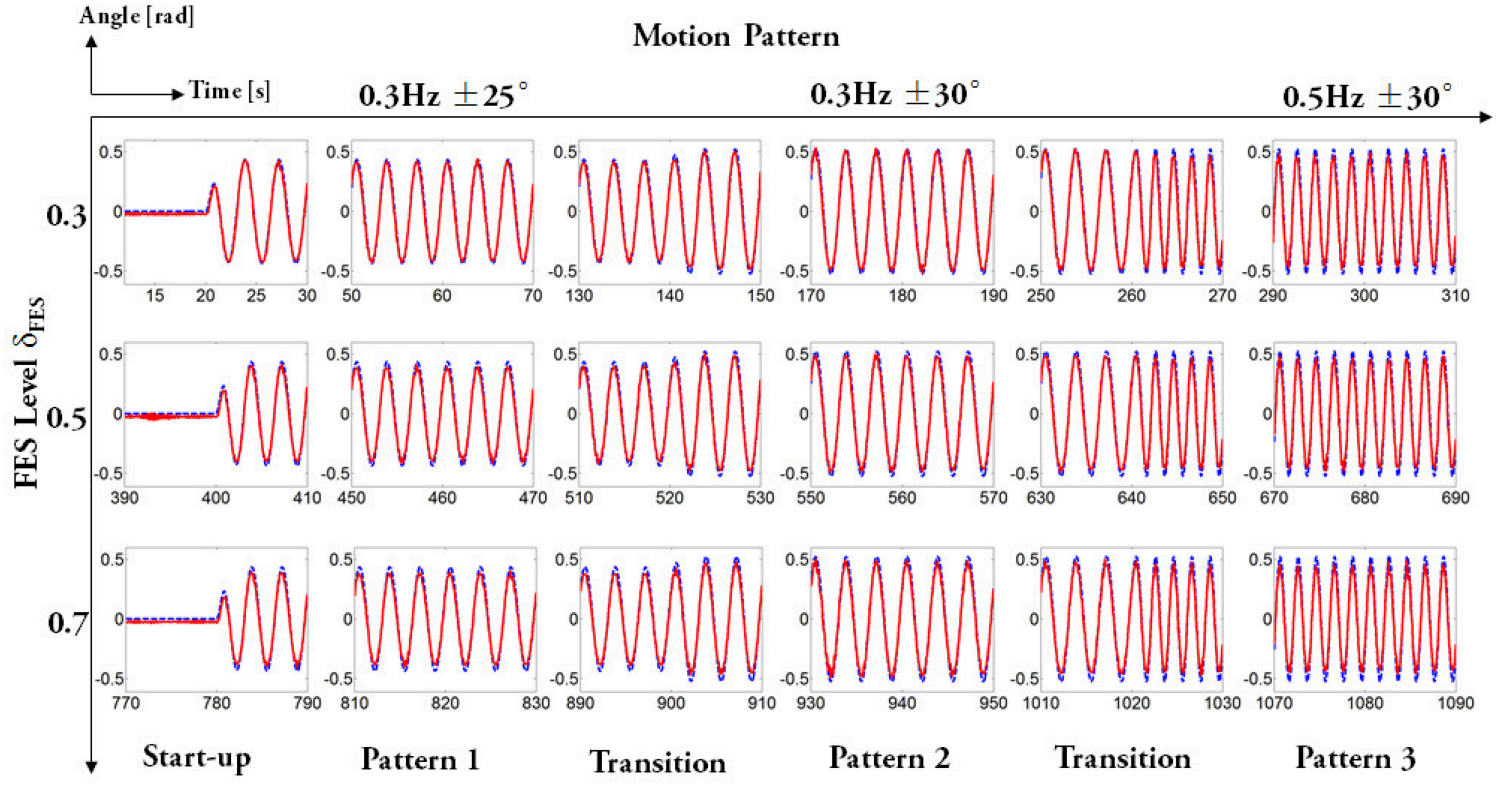
Trajectory tracking of P2 during the overall experimental procedure. For each subfigure, the x axis is time and the y axis is joint angle. The whole evaluation experiment contained three sessions determined by FES levels, and for each session, subjects underwent a continuous procedure including three kinds of motion patterns. The dashed lines represent desired trajectories and solid lines represent actual joint angle.

First of all, to obtain an intuitive view of the testing performance on FEXO Knee, arbitrarily the joint trajectories of the patient P2 during overall experimental procedure were presented in Figure 5. The good tracking performance between desired trajectory and actual knee joint angle is clearly observed. Especially, the trajectory is smooth and stable even during the transition periods between different motion patterns, which should be attributed to the merits of CPG.

**Figure 6:**
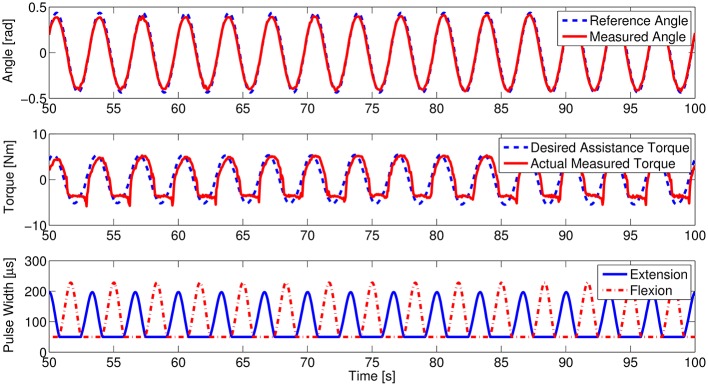
Real-time data of the healthy subject H3. The upper figure shows the trajectory tracking curves, the middle figure shows the assistive performance of the knee exoskeleton, and the lower figure shows modulated pulse width of two FES channels targeting quadriceps for extension and hamstrings for flexion.

To watch the performance including trajectories, torques, and controlled variables of FES in detail, the related real-time data of the healthy subject H3 under motion pattern 1 with the FES distribution gain at 0.3 were arbitrarily selected to show in Figure 6. We can see that the tracking performance is satisfactory, and the assistive effect of the knee exoskeleton (mutual torque) matches the desired values well. It demonstrates the efficiency of FEXO Knee, which can distribute the torque upon any requirements and keep FES-induced muscles and the exoskeleton work in a synchronized manner.

**Figure 7:**
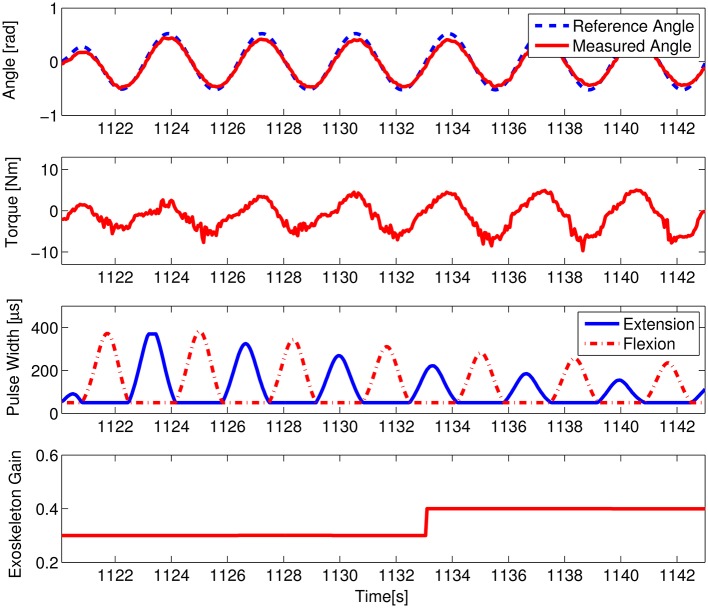
System performance in case of muscle force decline on the healthy subject H2. The first figure shows the trajectory tracking curves, the second figure shows the measured maximum mutual torque, the third shows the modulated pulse width of the two FES channels, and the fourth shows the variations of the exoskeleton torque distribution gain.

In our experimental paradigm, the interactive force sensors did not catch obvious muscle force decline due to muscle fatigue because of the simple swing motion without much effort and the short time for muscle stimulation. To imitate the muscle fatigue condition, we added an experiment to check the system performance in condition of muscle force decline. The real-time data on the healthy subject H2 are shown in Figure 7. When the muscle stimulation intensity was lowered in pre-setting program, the torque distribution gain (δ_*exo*_) of the exoskeleton automatically increased. It means the exoskeleton adaptively compensated the torque needed, i.e., the measured mutual torque also increased accordingly. The self-adaption of the system is mainly due to the function of the parameter regulator. The error between desired assistive torque and actual mutual torque updated the exoskeleton gain (δ_*exo*_) as shown in Equation (12). Please note that the updating time window for δ_*exo*_ is three cycle periods.

Five measures were proposed and analyzed, which could comprehensively evaluate the control performance of our system: (1) the absolute maximum amplitude of joint angle, (2) the duration of a single trial (i.e., a cycle period of knee joint movement), (3) the absolute maximum mutual torque between shank and exoskeleton, (4) the averaged error between reference trajectory of the exoskeleton and actual knee angle; (5) the averaged error between the desired assistive torque and measured mutual torque. The measures (1) and (4) indicates the position control performance. The measure (2) indicates the speed control performance. The measures (3) and (5) indicate the torque control performance. According to the experimental paradigm, there were totally nine conditions with different stimulation levels and motion patterns. The data during steady trials were extracted for evaluation and analysis, i.e., the trials 6~35 were segmented for the healthy subjects and the trials 6~25 for the patients. Each trial started from the zero position of knee joint. The data processing and statistical analysis were conducted in MATLAB (MathWorks, Natick, MA).

The experimental results regarding five measures (maximum angle, trial duration, angle error, torque error, and maximum mutual torque) are shown in the figures (**Figures 9**, **10**). The grand-averaged results of five healthy subjects and four patients are given separately. For every sub-figure, the lateral axis denotes the trial number (30 trials for healthy subjects, and 20 trials for hemiplegic subjects), and the solid lines represent the mean values across subjects and the shadow regions represent the standard errors of the mean values (±s.e.m) among subjects.

The statistical analysis was conducted to evaluate the general performance over the five healthy subjects and hemiplegic patients, respectively. The raw data were divided into trials, and each trial was equal to a complete cycle of knee extension and flexion (30 trials for healthy subjects, and 20 trials for hemiplegic subjects).

Based on the experimental protocol as shown in Figure 4, there are two factors (motion pattern and FES level). A two-way ANOVA including two factors (motion pattern and FES level) was applied firstly, and the results showed no significant interaction between the two factors (*p* = 0.96 > 0.1 for healthy subjects and *p* = 0.99 > 0.1 for paralyzed patients). As we focused on the factor of FES level, a one-way ANOVA was used to evaluate the performance further. Maximum angle, trial duration, and maximum mutual torque are key measures, which can show the steady and adaptation performance of the system under different FES levels (δ_*FES*_). In a single motion pattern, we checked the difference of the three measures among three FES levels.

**Figure 8:**
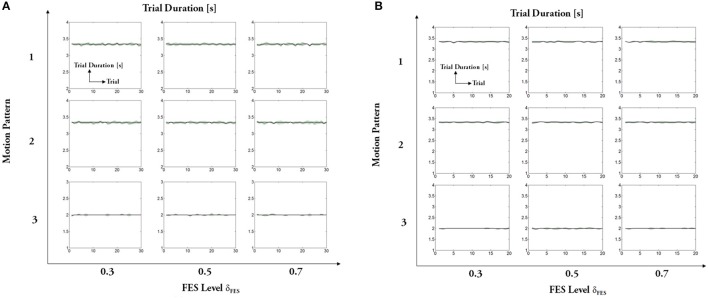
Trial duration: **(A)** healthy subjects, **(B)** hemiplegic patients. For each subfigure, x-axis indicates the trial number and y-axis indicates the trial duration.

**Figure 9:**
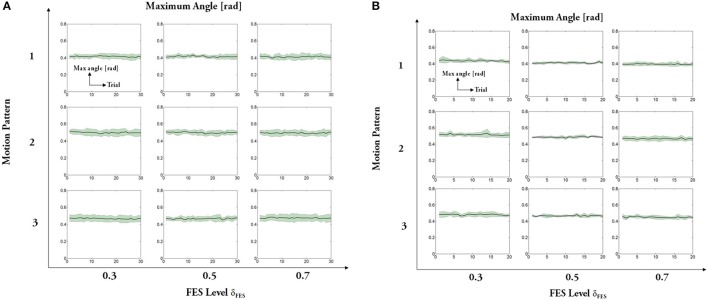
Maximum amplitude of joint angle: **(A)** healthy subjects, **(B)** hemiplegic patients. For each subfigure, x-axis indicates the trial number and y-axis indicates the absolute maximum angle. Solid lines represent the mean values across all subjects and shadow regions represent the standard error of the mean (±s.e.m.), similarly hereinafter.

Regarding statistical analysis on trial duration (see Figure 8), the FES level depending on torque distribution ratio is the unique factor. The ANOVA results do not show significant difference for the healthy subjects in different FES levels [motion pattern 1: *F*_(2, 87)_ = 0.05, *p* > 0.1; motion pattern 2: *F*_(2, 87)_ = 0.22, *p* > 0.1; motion pattern 3: *F*_(2, 87)_ = 0.04, *p* > 0.1], as well as the patients [motion pattern 1: *F*_(2, 57)_ = 0.01, *p* > 0.1; motion pattern 2: *F*_(2, 57)_ = 0.02, *p* > 0.1; motion pattern 3: *F*_(2, 57)_ = 0.39, *p* > 0.1]. It reveals that the variations of FES levels do not influence the trial duration of FEXO Knee in the same motion pattern. In other words, the swing frequency (motion speed) is steady, which is the desired merit for hybrid rehabilitation systems. Similarly, the ANOVA results show that variations of different FES levels do not have significant impact on actual maximum joint angle (see Figure 9). These results reflect that FEXO Knee can provide stable assistance for users. Even if FES is changing, the exoskeleton part can compensate the change in time, and achieve the desired motion features smoothly. However, in the same motion pattern, the maximum joint angle of paralyzed patients cannot keep a stale value in three FES levels, and there is a slight decrease as FES level increases (cf. Figure 9), which may be caused by the pathological conditions of muscles in the patients (weakness, atrophy, and rigid, etc.). When the FES level gets higher, the actual torque generated by patients' muscles is lower than expected, and the rigid knee joint prevents the exoskeleton from providing enough compensative torque, therefore, maximum joint angle cannot be fully reached. The actual torque generated by patients was smaller than healthy subjects, due to the weaker muscle activation of patients and the fact that the regulation of FES output depends on an open-loop control method. Nevertheless, even though some rough models (IDM, inverse muscular model, etc.) are used in the control scheme, the motion performance of FEXO Knee is satisfactory and no subjects have reported conflicted interaction between FES and the exoskeleton, which demonstrates the efficiency of the cooperative control strategy.

**Figure 10:**
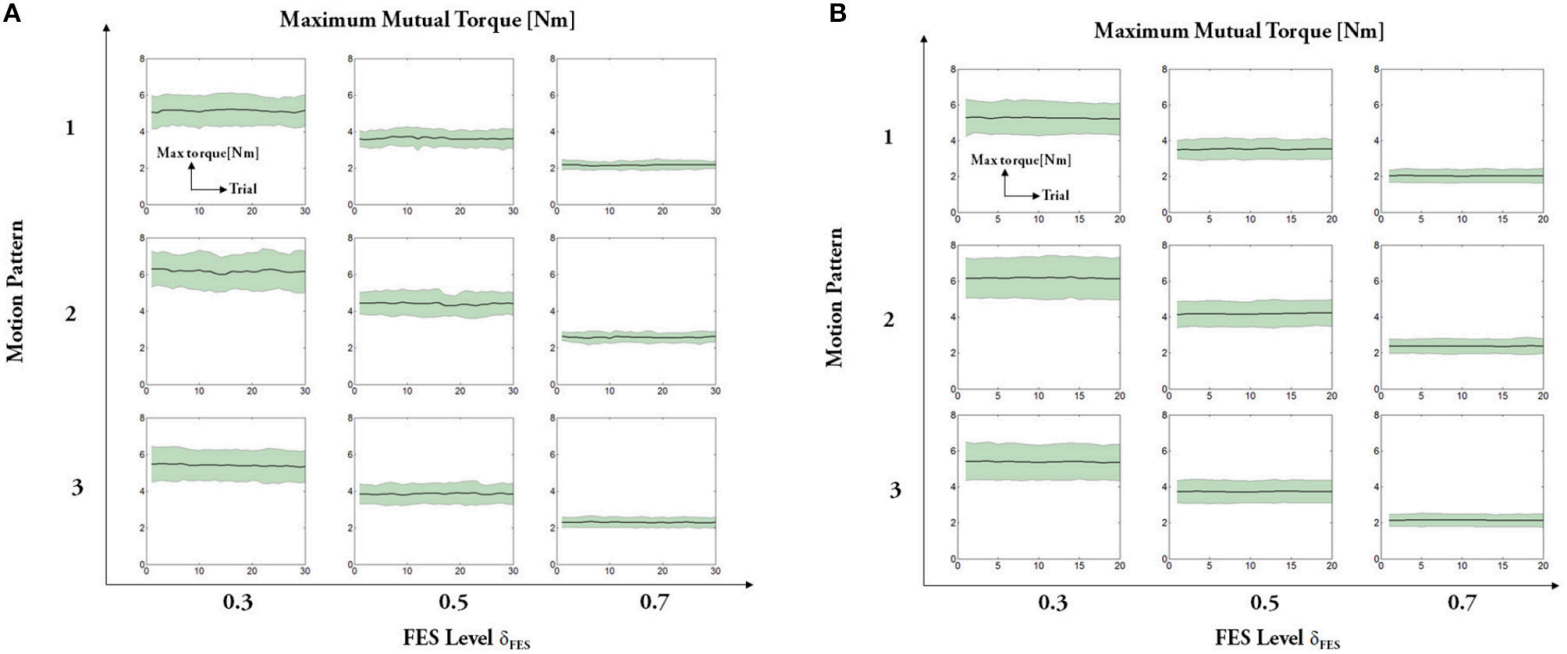
Absolute maximum mutual torque: **(A)** healthy subjects, **(B)** hemiplegic patients. For each subfigure, x-axis indicates the trial number and y-axis indicates the measured maximum mutual torque between shank and exoskeleton.

Regarding the absolute maximum mutual torque (see Figure 10), we can see that the assistive torque provided by the exoskeleton (measured mutual torque) declines as human muscles under FES generates larger force. The ANOVA results of the maximum mutual torque among different motion patterns are significant for healthy subjects [motion pattern 1: *F*_(2, 87)_ = 31,696, p<0.01; motion pattern 2: *F*_(2, 87)_ = 30,417.54, *p* < 0.01; motion pattern 3: *F*_(2, 87)_ = 58,308.27, *p* < 0.01], as well as the patients [motion pattern 1: *F*_(2, 87)_ = 78,032.9, *p* < 0.01; motion pattern 2: *F*_(2, 87)_ = 131,176.09, *p* < 0.01; motion pattern 3: *F*_(2, 87)_ = 131,562.45, *p* < 0.01]. The results reveal that the primary goal of FEXO Knee that aims to regulate the torque distribution between FES and the exoskeleton is accomplished to some extent.

**Figure 11:**
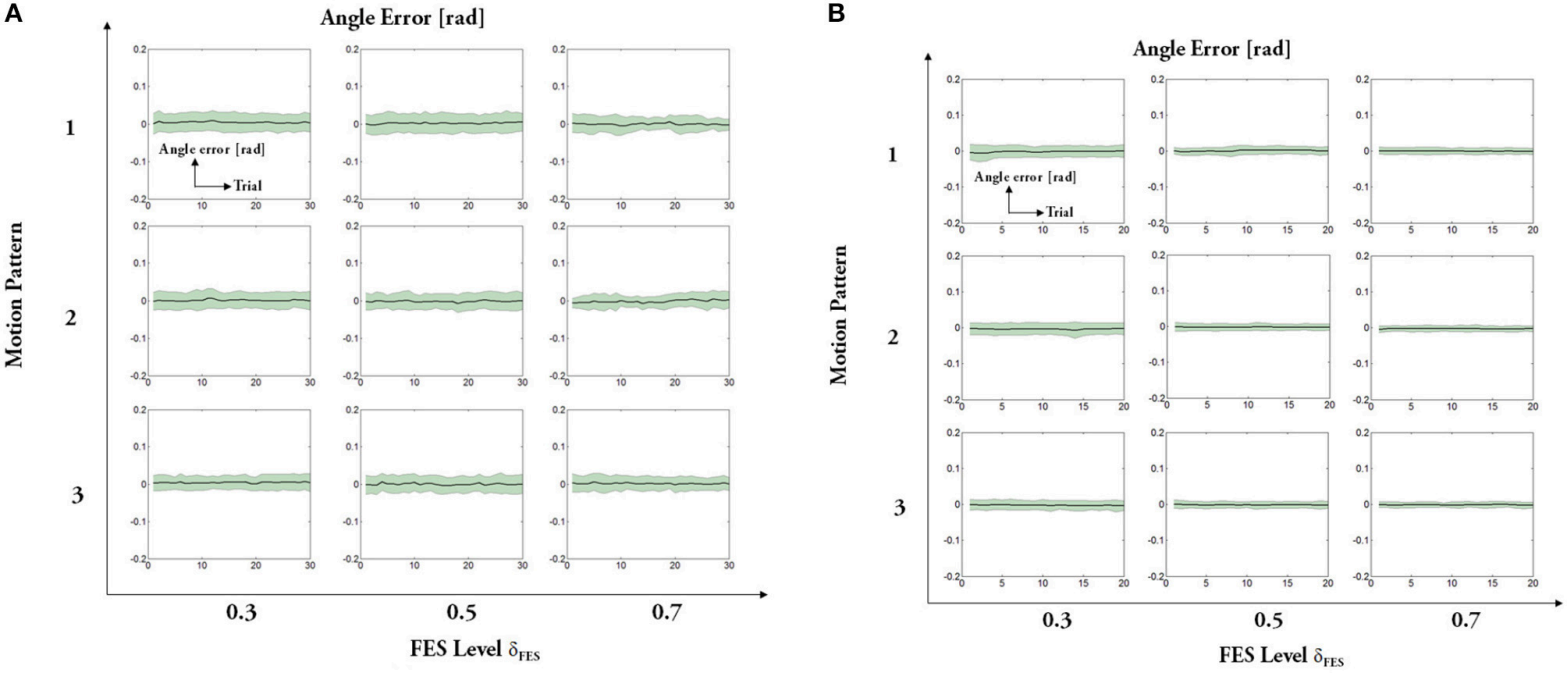
Averaged error between the reference trajectory and the actual knee angle: **(A)** healthy subjects, **(B)** hemiplegic patients. For each subfigure, x-axis indicates the trial number and y-axis indicates the averaged angle error.

**Figure 12:**
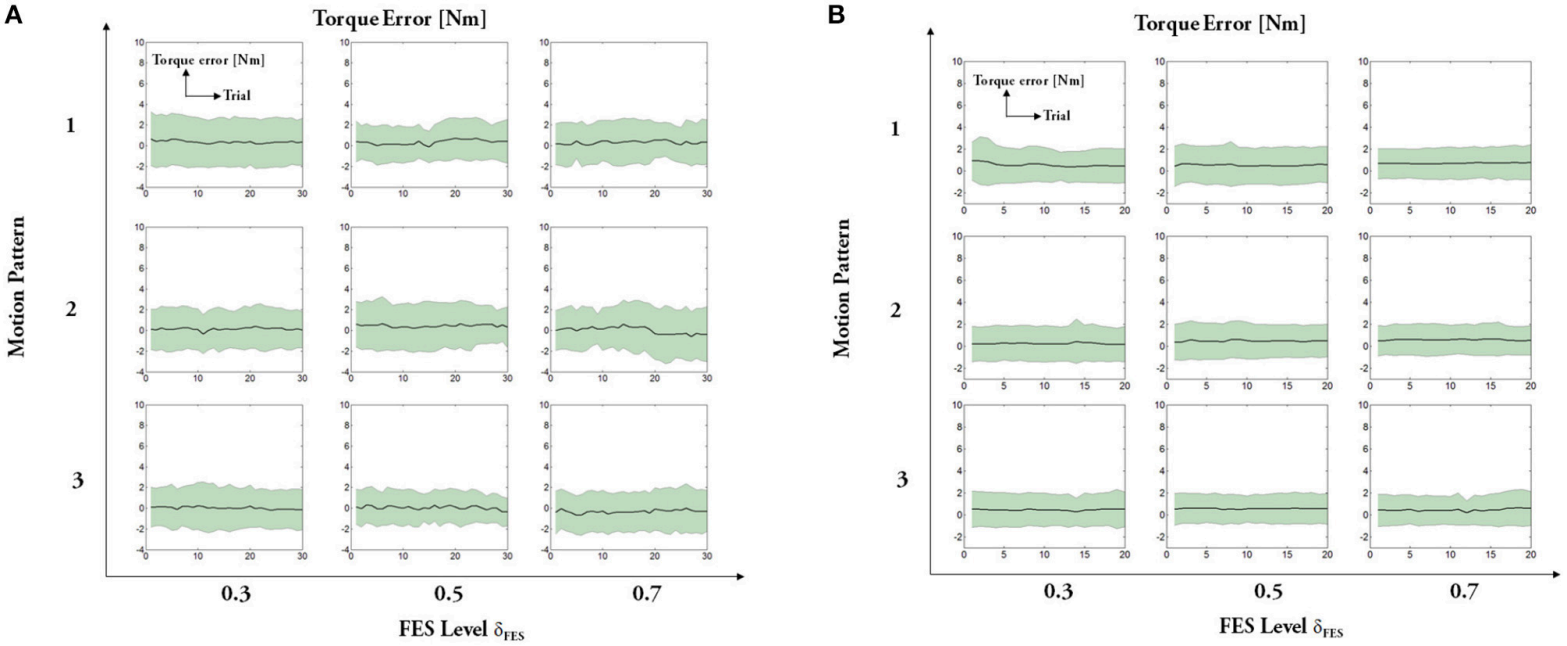
Averaged error between the desired assistive torque and the measured mutual torque: **(A)** healthy subjects, **(B)** hemiplegic patients. For each subfigure, x-axis indicates the trial number and y-axis indicates the averaged torque error.

Figures 11, 12 present results of other two measures: angle error (errors between the desired joint angle and the measured joint angle) and torque error (errors between the desired assistive torque and the measured mutual torque). The results reveal that the angle errors are kept in a relatively small range. From the results, we can also see that the averaged torque errors are limited to ±3 Nm, even if there are relatively large individual variations among the subjects. Given the fact the system does not seek the perfect torque tracking, the results are acceptable.

## 4. Discussion

In this paper, a novel approach based on cooperative control was introduced for exploring hybrid FES-exoskeleton rehabilitation. A self-made hybrid rehabilitation device called FEXO Knee was developed as the experimental platform. The exoskeleton has a compliant mechanism driven by a rotatory elastic actuator, and this is a highlight of the system. Series elastic actuators (SEAs) possess some specific advantages compared with traditional rigid actuators, including tolerance to abrupt force shock, capacity of energy storage and release, and steady force control, etc. (Pratt and Williamson, 1995; Yu et al., 2015). Actually, the SEAs do not seek absolute accuracy of tracking position trajectory but compliant human-robot interaction, which is more important in human-machine system.

The muscle nonlinearity and the exoskeleton compliance would bring about phase confliction problems. Therefore, we used a CPG network based on modified phase oscillators capable of online adjusting the phase difference to avoid possible out-of-phase trouble. CPGs possess some merits, which are especially favorable for the requirements of cooperative control in our hybrid system. Firstly, the CPG in this work has the capacity to generate stable oscillation, so as to keep robust under transient and slight disturbance. The state variables of the modified phase oscillator model can converge to the desired values while the convergent velocity is determined by some tunable values (cf. Equations 2–4). Secondly, the CPG has smooth transition capacity between different rhythmic oscillations after receiving commands from the higher level controller. As for the modified phase oscillators, the desired state values can be changed to form variable rhythmic patterns, and ensure the transition is continuous and smooth, which is essential to joint trajectory generation. Finally, the basic units (i.e., nonlinear oscillators) of the CPG has the capacity of establishing a network by coupling. The second term of Equation (2) reflects the coupling effect among phase oscillators. The coupling can be used to make different oscillators keep stable phase differences. Furthermore, the CPG as a network composed by these phase oscillators theoretically can generate any periodical trajectories. In the control scheme of FEXO Knee, the two nonlinear oscillators keep an adaptive phase difference with the help of a parameter regulator to avert possible conflictions between human leg and exoskeleton. Through the parameter regulator, sensory feedback is incorporated into CPG, thus a fully coupled dynamic system is accomplished in a big closed loop, and entrainment of CPG with plant output can make the whole system work in a synchronized way.

An advantage of the cooperative control is the arbitrary distribution of torque contribution between FES and exoskeleton. The tunable gains are set in feedfoward control of FES and feedback control of exoskeleton, respectively. Even though a lot of advanced control methods have been developed in controlling FES-actuated limbs to track reference trajectories accurately in previous research (Zhang et al., 2007), this work just adopted the feedfoward controller for FES to generate modulated pulse width based on an inverse muscular model. The control scheme of FEXO Knee does not aim to accomplish accurate position tracking merely by FES, but use a compliant actuator of exoskeleton to compensate the insufficient torque instead. The hybrid rehabilitation paradigm can overcome the deficiencies of FES and the exoskeleton, while achieving mutual promotion of these two technologies. Therefore, a simple but practical method is enough for FES controller.

Previous studies have introduced mechanical actuators to assist FES to achieve locomotion including swinging, walking and cycling, but the mutual interaction between skeletal muscles and mechanical actuators were rarely considered. Most hybrid rehabilitation systems intended to either reduce mechanical power consumption (Ha et al., 2012) or minimize the resistive torque caused by mechanical actuation (del Ama et al., 2014). It should be emphasized that the interactive force sensors are implemented in FEXO Knee. Based on the force sensors, the arbitrary torque distribution can be realized via the cooperative control. In our control strategy, the coordination between FES and the exoskeleton is ascribed to the torque distribution gain that denotes their actuating effort for limb locomotion, and the possible confliction is solved by adjusting the phase difference of their reference trajectories. It differs from the previous control methods in which muscular and mechanical actuation have separate control objectives. For example, Hunt et al. (2004) proposed an integrated control strategy containing two closed loops for hybrid FES cycling, which provided feedback control of leg power output (via automatic adjustment of stimulation intensity) and cycling cadence (via electric motor control), respectively. In fact, FEXO Knee aims to accomplish a general goal (desired angular position) while allotting the workload (assistive torque) between FES and exoskeleton in arbitrary ratio, and this task is realized by the cooperative control.

Some evaluation experiments of FEXO Knee were conducted on both healthy subjects and hemiplegic patients. Different levels of FES were applied on the subjects, and the statistical analysis on the experimental data revealed that the cooperative control method could balance the effort of FES and the exoskeleton. Besides, the variations of stimulation strength did not influence the movement performance, i.e., the angular position and duration of each trial did not change, which verified the adaptability and robustness of the system. In clinical application, the cooperative control method used for dealing the torque distribution between FES and exoskeleton would be very helpful for patients.

The experimental results have shown the performance is satisfactory for both healthy subjects and paralyzed patients, and none reported significant confliction between leg and exoskeleton, which demonstrated the efficiency of the cooperative control strategy. FEXO Knee kept stable swing motion exhibited by the amplitude and period of joint motion, while the exoskeleton could adaptively compensate the insufficient part of necessary torque for shank swing, i.e., the mutual torque was changed accordingly (see Figure 10). The patients had pathological muscular conditions including weakness, atrophy, rigidness, and so on, so the actual torque generated by muscles of patients are lower than expected under FES. While the closed-loop control of exoskeleton could detect the torque error and position error, and thus automatically provided enough compensative torque to accomplish the desired motor pattern. In real FES clinical rehabilitation, paraplegic patients usually need to walk bearing their body weight for some time, so continuous and intensive electrical stimulation can cause significant muscle fatigue. In our experimental paradigm, the muscle fatigue phenomenon is not obvious because of the simple swing motion without much effort and short time for muscle stimulation. However, the proposed strategy is capable of dealing with muscle force decline like the situation of muscle fatigue. The parameter regulator can update the tunable gain (τ_*exo*_) online, which works together with the closed-loop control mechanism of exoskeleton, is a trump card.

In future, some work should be conducted to improve the FEXO Knee system with cooperative control. The feedfoward controller for FES is based on a rough inverse model of human muscles under electrical stimulation, which still needs manual system identification. Some intelligent methods such as artificial neural networks may be used to accomplish automatic parameter identification facing individual variability, especially for paralyzed patients (Chang et al., 1997; Kurosawa et al., 2005). The FEXO Knee mainly focuses on rhythmic movements of knee joint, and we need extend its applications toward complex lower-limb movements such as walking. Therefore, a hybrid FES-exoskeleton system with multiple degrees of freedom is expected to be developed.

## 5. Conclusions

This paper presents a novel cooperative control scheme for better human-machine interaction and physical rehabilitation in a hybrid FES-exoskeleton system called FEXO Knee. Torque distribution between the two kinds of actuators (muscles under electrical stimulation and electrical motor with SEA) is regulated via tunable gains. A CPG network containing two modified phase oscillators generates reference motion of FES and the exoskeleton. Cooperative control adaptively adjusts the phase difference between the two oscillators to avoid unexpected conflictions between the two compliant mechanisms, allowing better interaction. The control method provides an effective solution for dealing with the coordination between FES and the exoskeleton in a hybrid system. The performance has been testified by some evaluation experiments on both healthy subjects and hemiplegic patients. We believe the FEXO Knee system for physical rehabilitation would be very promising for paraplegic patients to restore the extremity function.

## Author contributions

DZ and WX conceived of the study and designed the experiments. YR, KG and DZ designed the system, conducted the experiment, and analyzed the data. JJ and WX performed the clinical test on the patients. DZ and YR drafted the manuscript.

### Conflict of interest statement

The authors declare that the research was conducted in the absence of any commercial or financial relationships that could be construed as a potential conflict of interest.
